# The role of REM sleep theta activity in emotional memory

**DOI:** 10.3389/fpsyg.2015.01439

**Published:** 2015-10-01

**Authors:** Isabel C. Hutchison, Shailendra Rathore

**Affiliations:** ^1^School of Psychological Sciences, Faculty of Medical and Human Sciences, University of Manchester, Manchester, UK; ^2^Neuroscience, Physiology and Pharmacology, University College London, London, UK; ^3^Centre of Mathematics and Physics in the Life Sciences and Experimental Biology, University College London, London, UK

**Keywords:** REM sleep, emotional memory, encoding, acetylcholine, habituation, theta activity

## Abstract

While non-REM (NREM) sleep has been strongly implicated in the reactivation and consolidation of memory traces, the role of rapid-eye movement (REM) sleep remains unclear. A growing body of research on humans and animals provide behavioral evidence for a role of REM sleep in the strengthening and modulation of emotional memories. Theta activity—which describes low frequency oscillations in the local field potential within the hippocampus, amygdala and neocortex—is a prominent feature of both wake and REM sleep in humans and rodents. Theta coherence between the hippocampus and amygdala drives large-scale pontine-geniculo-occipital (PGO) waves, the density of which predicts increases in plasticity-related gene expression. This could potentially facilitate the processing of emotional memory traces within the hippocampus during REM sleep. Further, the timing of hippocampal activity in relation to theta phase is vital in determining subsequent potentiation of neuronal activity. This could allow the emotionally modulated strengthening of novel and gradual weakening of consolidated hippocampal memory traces during REM sleep. Hippocampal theta activity is also correlated with REM sleep levels of achetylcholine - which is thought to reduce hippocampal inputs in the neocortex. The additional low levels of noradrenaline during REM sleep, which facilitate feedback within the neocortex, could allow the integration of novel memory traces previously consolidated during NREM sleep. We therefore propose that REM sleep mediates the prioritized processing of emotional memories within the hippocampus, the integration of previously consolidated memory traces within the neocortex, as well as the disengagement of consolidated neocortical memory traces from the hippocampus.

## Introduction

Though the body may seem inert during sleep, the brain most definitely is not. Mammalian sleep cycles through multiple electrophysiologically and neurochemically distinct sleep stages. These stages are generally split into two categories, based on the occurrence of rapid-eye movements (REMs), i.e., REM and non-REM (NREM) sleep. While evidence strongly supports a pivotal role of NREM sleep in memory consolidation, the function of REM sleep remains elusive.

In this review, we propose that REM sleep represents a unique brain state that allows the emotionally modulated integration and recombination of neocortical memory traces previously consolidated during NREM sleep. In addition, we suggest that REM sleep is involved in the gradual disengagement of successfully consolidated memory traces from the hippocampus—thus mediating the decontextualization of novel memories, allowing generalization, abstraction, etc. To support this, we initially review behavioral evidence linking REM sleep and emotional memory processing in both rodents and humans. We then discuss how this relationship may be mediated by electrophysiological (in particular theta) activity within the hippocampus, amygdala and neocortex during REM sleep.

## REM vs. NREM Physiology in Humans and Rodents

REM and NREM sleep differ remarkably in several ways: while NREM sleep is characterized by high amplitude, low frequency (0.3–4 Hz) electroencephalographic (EEG) activity reflecting synchronization across large neuronal populations, the low-amplitude, mixed frequency EEG activity observed during REM sleep more closely resembles that of quiet wake (Llinas and Ribary, 1993; Steriade et al., 1996). Neuromodulator levels also differ between these two brain states: during NREM sleep, acetylcholine levels in the brain stem, forebrain, and hippocampus are at a physiological nadir (Hobson et al., 1975; Marrosu et al., 1995), while cholinergic modulation during REM sleep increases to levels just below that of wake in the neocortex and even exceeds wake levels in the hippocampus (Hasselmo, 1999). In contrast, while aminergic (i.e., serotonergic and noradrenergic) neurons fire at reduced rates during NREM sleep compared to wake (Aston-Jones and Bloom, 1981), they are almost completely silenced during REM sleep (Pace-Schott and Hobson, 2002). Besides the occurrence of REMs, REM sleep is also identified by a significant reduction in muscular tone. This atonia of the skeletal musculature is a vital characteristic of REM sleep, the loss of which results in dream enactment (McCarter et al., 2012). Furthermore, while dreams can occur during all sleep stages, those of REM sleep tend to have a comparatively bizarre, emotional, and vivid quality (Suzuki et al., 2004).

In humans, NREM sleep is divided into light sleep and slow-wave (or deep) sleep (Iber et al., 2007), while in rodents all NREM sleep stages are collectively referred to as NREM or slow-wave sleep (SWS; van Twyver, 1969; Genzel et al., 2014; Oyanedel et al., 2014), with only few studies differentiating between light and deep NREM sleep (e.g., Benedetto et al., 2013), and in some cases including a spindle-rich transition to REM sleep (TR) phase (e.g., Watts et al., 2012) which is not reported in the human literature. In rodents and humans, the proportion of SWS in a given sleep cycle decreases with diminishing sleep pressure (Borbély and Achermann, 1999; Yasenkov and Deboer, 2012). In human nocturnal sleep, SWS predominates during the first half of the night while REM sleep—regulated by circadian factors—displays an inverse relationship with SWS, becoming increasingly prevalent toward the morning (Wurts and Edgar, 2000). In rodents, REM sleep does not appear to follow a circadian rhythm (Yasenkov and Deboer, 2012) and takes up a higher proportion of overall sleep compared to humans (Mendelson and Bergmann, 1999).

## NREM Sleep and Memory Consolidation

The idea that sleep is important in the consolidation and processing of both recent and remote memories is well established (for an extensive review, see Rasch and Born, 2013). Based on early rodent work this memory function of sleep was primarily ascribed to REM sleep. However, more recent work in both rodents and humans strongly supports a role of NREM sleep in memory reactivation and consolidation (Stickgold, 2005; Girardeau et al., 2009; Diekelmann and Born, 2010; Ego-Stengel and Wilson, 2010; Rasch and Born, 2013). The high frequency thalamocortical spindles and associated hippocampal sharp-wave ripples, which occur during both stage 2 NREM and SWS (Genzel et al., 2014), are thought to reflect processes underlying synaptic plasticity (Steriade, 1999; Sejnowski and Destexhe, 2000). The low levels of acetylcholine during NREM sleep disinhibit communication between the hippocampus and neocortex (Hasselmo, 1999). This, in conjunction with the high amplitude slow waves of SWS is thought to drive the transfer of declarative memory traces from the hippocampus to the neocortex (Rasch and Born, 2013) by providing windows of wide-spread depolarization during which higher frequency activity (including spindles) is synchronized across various brain regions (Battaglia et al., 2004; Compte et al., 2008; Mölle and Born, 2009, 2011). In support of this, overnight improvement in memory performance is predicted by the amplitude of slow waves in both rats and humans (Marshall et al., 2004; Heib et al., 2013; Binder et al., 2014), as well as the occurrence of spindle activity during the up-phases of slow oscillations in humans (Mölle et al., 2011; Cox et al., 2012; Ngo et al., 2013). Similarly, the duration of stage 2 NREM sleep predicts overnight consolidation of both declarative and motor memories (Walker et al., 2002; Fogel and Smith, 2006; Ruch et al., 2012). Studies investigating the function of REM sleep have been comparatively unfruitful—often yielding conflicting results, thus leading to an overall neglect of this sleep stage. There is however considerable evidence linking REM sleep with the processing of emotional memories, discussed in the following section. A separate line of research implicates REM sleep in the consolidation of procedural skills, the mechanism of which deserves consideration; however this exceeds the scope of this review.

## REM Sleep and Emotional Memory

Events that elicit an emotional response tend to be remembered more reliably and more long-term than comparatively unemotional events (LaBar and Cabeza, 2006). Emotional responses are most commonly elicited by situations relevant to survival. Although emotions are undoubtedly important in guiding immediate behavior—whether by triggering a fight or flight response, driving reproduction or seeking nourishment—retaining memory of the experience that elicited the emotion carries the additional benefit of guiding future behavior in similar situations, and thus would improve overall chances of survival (Hamann, 2001). The neural mechanism underlying the influence of emotion on long-term memory retention involves co-activation of the hippocampus and the amygdala—the emotional center of the brain. The amygdala appears to modulate hippocampal activity, thus facilitating the preferential encoding of emotional memories and potentially their tagging for future consolidation.

Several studies support the central role of the amygdala in mediating the prioritized consolidation of emotional vs. neutral memories. Bilateral amygdala damage selectively impairs emotional memory (Adolphs et al., 1997). In a separate study, the degree of left hippocampal damage was found to negatively predict emotional memory performance (Richardson et al., 2004). More strikingly, amygdala activity during memory encoding predicted later recall of negative (Cahill et al., 1996) and positive (though not neutral) memories (Hamann et al., 1999). Furthermore, using event-related fMRI, Dolcos et al. (2004) showed that the interaction of activity between the amygdala and hippocampus predicted recall of emotional vs. neutral memories.

In rodents, memory tasks tend to be inherently emotional. Often the emotional response represents the actual memory (e.g., in fear conditioning or extinction), or else it is used as an incentive to perform a memory task (e.g., a food reward or the avoidance of pain). Similarly to the work in humans, there is a large body of evidence supporting a role of amygdala activity in emotional memory (i.e., fear conditioning) in rodents (for a review, see LeDoux, 2003). Though—due to the lack of an adequate non-emotional control—the role of the amygdala in more indirect forms of emotional memory in rodents is less clear.

Sleep appears to facilitate the preferential consolidation of emotional memories. Though there is evidence supporting a role of SWS in this process (Groch et al., 2011; Cairney et al., 2014), a larger body or research implicates REM sleep in both the selective strengthening of emotional memories as well as the modulation of the emotional response associated with specific stimuli. This dual-process is described in the “sleep to forget sleep to remember” (SFSR) hypothesis proposed by Walker (2009). Evidence for the role of REM sleep in emotional memory processing is summarized in the following two sections.

### Behavioral Evidence

Early studies on REM sleep examined the effects of REM sleep deprivation (REMSD) on memory consolidation and encoding in rodents. Post-training REMSD was consistently found to impair avoidance learning (Pearlman, 1969; Leconte and Bloch, 1970; Fishbein, 1971; Smith and Kelly, 1988), avoidance conditioning (Leconte and Bloch, 1970), and fear conditioning (Menz et al., 2013). Conversely, REMSD preceding training impeded the efficiency of fear conditioning (McGrath and Cohen, 1978; Smith, 1985; Bueno et al., 1994) and avoidance learning (Hartmann and Stern, 1972; Sagales and Domino, 1973; Danguir and Nicolaidis, 1976). Fear extinction was also impaired following REMSD compared to uninhibited sleep in rats (Silvestri, 2005; Fu et al., 2007). However, it appears the effects of REMSD are only short-lived, not persisting beyond recovery sleep (Fishbein, 1971). Furthermore, the deleterious effects of REMSD are likely to be explained by the extreme stress resulting from the so-called flowerpot method used to prevent REM sleep (Horne and McGrath, 1984). This method typically involves placing the animal on a small platform above water so that when REM sleep associated muscle atonia sets in, the animal slips into the water and awakens.

A recent study using REMSD provides more striking evidence for a role of REM sleep in emotional memory processing: Ravassard et al. (2015a) trained rats in contextual fear conditioning followed by short-term and non-stressful REMSD. They found that 4 h of REMSD impaired both the consolidation of contextual fear conditioning and long-term potentiation (LTP) within the CA1 region of the hippocampus. Conversely, rodents that obtained a comparably higher amount of REM sleep following contextual fear conditioning displayed stronger consolidation, as well as greater hippocampal LTP. Furthermore, both measures were positively correlated with REM sleep amount. This fits with further evidence showing that increasing REM sleep duration in rats—through either carbachol (an acetylcholine agonist) or through REMSD-induced REM sleep rebound—led to enhanced memory retention of a reward-memory based Y-maze task across sleep (Wetzel et al., 2003)—suggesting REM sleep may also benefit positive emotional memory processing.

In humans, sleep in general has been shown to benefit fear extinction (Pace-Schott et al., 2009; Kleim et al., 2014). The amount of REM sleep obtained following fear extinction was shown to predict a decrease in autonomic arousal based on skin conductance (Spoormaker et al., 2010). Conversely, disrupting sleep through repeated awakening only impaired extinction if awakenings occurred during REM sleep, but not if they occurred during NREM sleep (Spoormaker et al., 2012). In addition to supporting fear extinction, REM sleep was found to predict post-sleep recognition of negative emotional pictures (Nishida et al., 2009; Groch et al., 2013), negative and positive emotional faces (Wagner et al., 2007) as well as recall of emotional texts (Wagner et al., 2001) compared to neutral controls. Administration of hydrocortisone during sleep following an emotional memory task resulted in superior recognition for emotional vs. neutral images (van Marle et al., 2013). Although sleep was not recorded in this study, the observed strengthening of emotional memory could be related to cortisol-mediated processes during REM sleep, as cortisol levels are naturally elevated during REM compared to NREM sleep (Steiger, 2007).

### Physiological Evidence

Physiological evidence also supports a role of REM sleep in memory. Areas implicated in memory processing during wake, in particular limbic circuits within the medial temporal lobe, are highly active during REM sleep (Maquet et al., 1996; Braun, 1997; Nofzinger et al., 1997; Braun et al., 1998; Nir and Tononi, 2010). At a cellular level, Pavlides and Winson (1989) observed reactivations of hippocampal neurons active during prior wakefulness during subsequent REM sleep. Even at a molecular level—plasticity-related gene expression increases within the hippocampus during REM sleep (Ribeiro et al., 1999). A recent study from the same group compared mRNA levels of plasticity-related genes within the hippocampus following either exposure to a novel or familiar control environment (Calais et al., 2015). The rats were killed either after 30 min of stable wake, SWS or REM sleep. mRNA expression of several plasticity-related genes were significantly upregulated during REM sleep following exposure to a novel environment—though not during post-training SWS or wake. There was also no upregulation of plasticity-related gene expression in rats who had not been exposed to the novel environment. A further recent study also revealed that increasing REM sleep amount through REMSD-induced rebound up-regulated the expression of plasticity-related transcription factors within the hippocampus (Ravassard et al., 2015b).

A distinct role for REM sleep in memory is also supported by the striking similarities and contrasts between neuromodulator states in REM sleep and wakefulness. The wake-like levels of acetylcholine in the limbic system suppress excitatory feedback potentials within the hippocampus and in the cortex (Hasselmo and Bower, 1993). During wake, this is thought to promote memory encoding by allowing the formation of new memory traces within the hippocampus without interference from previously stored memory traces (Hasselmo, 2006). Noradrenaline has been shown to suppress excitatory feedback transmission within the somatosensory and piriform cortex (Dodt et al., 1991; Hasselmo et al., 1997; Hasselmo, 1999), but not the hippocampus (Mueller et al., 1981). While wake is characterized by both high acetylcholine and noradrenaline levels, in REM sleep only acetylcholine is raised. As a consequence, excitatory feedback within the neocortex would remain uninhibited during REM sleep (Hasselmo, 1999), while hippocampal afferent inputs would be suppressed (Marrosu et al., 1995).

Based on these neuromodulator states, it has been proposed that during REM sleep, memories within the neocortex—free from interference from the hippocampus—recombine and potentially integrate into existing memory networks between periods of NREM sleep-dependent memory consolidation (Hasselmo, 1999; Walker, 2009; Walker and Stickgold, 2010; Sterpenich et al., 2014). It is important to stress that most of these observations are performed on cortical/hippocampal slices, thus the relationships inferred in terms of cortical-hippocampal interaction are very tentative.

### REM Sleep, an Emotional Brain State?

Rapid-eye movement sleep possesses a unique physiology which appears particularly amenable to the processing of emotional memories (Paré et al., 2002; Hu et al., 2006). Functional neuroimaging studies reveal significantly increased activation in the amygdala, striatum, hippocampus, medial prefrontal cortex and insula, which are areas strongly associated with emotional processing in wake (Nofzinger, 2005; Miyauchi et al., 2009; Dang-Vu et al., 2010). The heightened activity within the limbic system in particular (Maquet et al., 1996; Wehrle et al., 2007; Miyauchi et al., 2009) alludes to the established link between limbic activation during emotional memory encoding and future recall (Cahill, 2000; McGaugh, 2004). Possibly as a consequence of its emotional physiology, REM sleep is unique for its comparably emotional dreams (Hobson et al., 2000) which often contain elements of the dreamer’s recent memories (Nielsen and Powell, 1992; van Rijn et al., 2015).

## REM Sleep and Emotional Arousal

In addition to a general role of REM sleep in emotional memory processing, a separate line of research has emerged concentrating on a more specific link between REM sleep and the modulation of emotional responses. Whether this role is part of the same mechanism, or relies on distinct processes is unclear and needs to be investigated more systematically. This section reviews evidence specifically linking emotional response modulation and REM sleep.

### The Co-Morbidity of REM Sleep and Mood Disorders

Though many psychological disorders are comorbid with sleep disorders, it is particularly of note that mood disorders tend to be associated with unusual REM sleep: in depression, REM sleep is pathologically increased (Tsuno et al., 2005; Armitage, 2007; Gottesmann and Gottesman, 2007); a hallmark of post-traumatic stress disorder (PTSD) is the occurrence of flashbacks during REM sleep—often resulting in dream enactment and distressing awakenings (Mellman et al., 2007); while in patients suffering from anxiety, REM sleep percentage and REM density during REM sleep tend to be reduced (Rosa et al., 1983; Fuller et al., 1997). Thus pathological REM sleep may underlie some of the symptoms of mood disorders (Walker and van der Helm, 2009). Even in mice models for depression (i.e., stress vulnerable or chronically stressed strains), REM sleep appears to be disinhibited (Kimura et al., 2014), suggesting that REM sleep is related to emotional processing in both humans and rodents.

### Experimental Evidence for a Role of REM Sleep in Emotional Regulation

Some evidence suggests a sleep-dependent decrease in both subjective emotional arousal and autonomic response to negative stimuli compared to an equally long period of wake in humans (Gujar et al., 2011; van der Helm et al., 2011). In line with this notion, sleep-dependent habituation was only observed across naps containing REM sleep, not across naps consisting solely of NREM sleep (Gujar et al., 2011).

In contrast, Groch et al. (2013) found that subjective ratings of arousal to negative images was preserved over both SWS-rich early and REM sleep-rich late night sleep using a split-night design. In a further study, participants rated emotional stimuli as more negative across late sleep compared to early sleep (Wagner et al., 2002). Similarly, subjective emotional arousal went down across wake and was maintained across sleep (Baran et al., 2012). The degree of arousal maintenance was associated with greater time spent in REM sleep. REM sleep amount also predicted an increase in autonomic response in the form of skin conductance to emotional images shown before and after sleep (Baran et al., 2012). Furthermore, REMSD reduced arousal ratings to negative images presented before and after sleep (Lara-Carrasco et al., 2009). Thus it appears that REM sleep may modulate emotional arousal, however the direction of this change may depend on other yet to be determined factors, such as the nature of the emotional stimuli, the stress experienced during the task or possibly the involvement of memory (Genzel et al., 2015).

## Theta Activity

Theta activity describes synchronized oscillating local field potentials of neuronal populations within the range of 4–10 Hz initially observed in rodents (Siapas et al., 2005). It is considered a characteristic of hippocampal activity during both active exploratory behavior and REM sleep (Winson, 1974; Kemp and Kaada, 1975; Buzsáki, 2002). Rodents also display theta activity within the amygdala and ventromedial prefrontal cortex (Sörman et al., 2011; Brankačk et al., 2012)—areas strongly associated with cognitive and affective functions (Siapas et al., 2005; Sigurdsson et al., 2010). Furthermore, theta activity can be synchronized across disparate brain regions in wake (O’Neill et al., 2013) and REM sleep (Popa et al., 2010). Although humans also display a distinct 4–10 Hz hippocampal activity during both active wake (Burgess and Gruzelier, 1997; Ekstrom et al., 2005; Lega et al., 2012) and during REM sleep (Cantero et al., 2003) which is also observed in the neocortex (Cape et al., 2000; Nishida et al., 2009), this activity does not appear to be synchronized between the hippocampus and neocortex (Cantero et al., 2003; Axmacher et al., 2008). Instead, a slower ∼3 Hz delta range activity—referred to as either rhythmic slow activity (RSA) or slow theta—has been proposed to be more physiologically analogous to rodent theta activity (Moroni et al., 2007; Lega et al., 2012). Similarly to the theta activity observed in rodents, human slow theta (hence forward referred to simply as theta activity) also occurs in the human hippocampus during both wake and REM sleep (Moroni et al., 2007; Lega et al., 2012). The possible explanation for humans having a slower version of theta activity is the larger brain size which may require slower oscillations to travel greater distances between brain regions (Moroni et al., 2007). Accordingly, a slower hippocampal theta activity is also seen in dogs, cats, and monkeys (Lega et al., 2012).

### Theta Generation and the Role of Acetylcholine in Rats

Hippocampal theta activity appears to originate from nuclei within the brain stem which project via the hypothalamus to the septal complex comprising of the medial septum and a subregion of the Broca area (Pignatelli et al., 2012). The septal complex, in turn, projects to the hippocampus via the fimbria-fornix pathway. The medial septum contains pacemaker cells which fire at theta frequency (Dragoi et al., 1999). Some of these pacemaker cells release acetylcholine (Mesulam et al., 1983) and GABA (Freund, 1989). Inhibiting medial septum cell activity though targeted injection of lidocaine (Winson, 1978) or muscimol (Bland et al., 1996) leads to the complete suppression of hippocampal theta oscillations. Both acetylcholine and GABA jointly contribute to generating theta, as reductions of either leads to partial but not complete abolishment of theta power (Yoder and Pang, 2005; Li et al., 2007).

Given the strong link between acetylcholine and theta activity, the role of acetylcholine in memory processes within the hippocampus is highly indicative of the function of theta activity. It appears that high levels of acetylcholine enhance memory encoding during wakefulness, yet do not affect retrieval in a range of learning tasks in both rats and humans (for a review, see Hasselmo, 2006). Early work in rats showed that blocking acetylcholine through muscarinic antagonists (such as scopolamine) disrupted memory encoding if the drug was administered prior to learning, as opposed to during the gap between learning and recall (Ghoneim and Mewaldt, 1975, 1977). In humans scopolamine also disrupted encoding of memories without affecting retrieval (Atri et al., 2004; Hasselmo and McGaughy, 2004). Thus it appears acetylcholine is only involved in the encoding though not the consolidation of novel hippocampal memory traces during wake.

This has enticing implications for investigating the function of REM sleep, during which hippocampal acetylcholine levels exceed those of wake. This would suggest encoding-related processes occur during REM sleep—in stark contrast to the acetylcholine-independent memory consolidation processes occurring during NREM sleep.

### The Role of Wake Theta in Memory

Hippocampal theta activity during wake has been associated with memory formation and function in a number of species (Dragoi and Buzsáki, 2006; Montgomery et al., 2008; Mizuseki et al., 2009). The specific role of theta activity in this is thought to be the binding of disparate brain regions during encoding and retrieval (Vertes, 2005).

Physiological evidence strongly supports a role of hippocampal theta activity in rats in the formation of novel memories during wake. Thus seminal *in vitro* work by Huerta and Lisman (1995) demonstrated that a priming pulse (four pulses delivered at 100 Hz) induces LTP in the hippocampal CA1 of a brain slice only if the pulse arrives at the peak of carbachol-induced theta activity (defined by the authors as 5–12 Hz). Conversely, pulses delivered at the negative peak of theta activity resulted in long-term depression (Huerta and Lisman, 1995; Hölscher et al., 1997). This observation, initially made in hippocampal slices, has been subsequently confirmed in wake behaving animals by stimulating at peaks/troughs of theta in the perforant path (Orr et al., 2001) and in CA1 (Hyman et al., 2003).

Electroencephalographic current source density data has shown that subregions of the rat hippocampus are out of phase with respect to theta activity (Buzsáki et al., 1986; Brankačk et al., 1993). Specifically, theta activity within the entorhinal cortex is 90°out of phase with that of CA3, and is in phase with that of CA1 cortex (Mizuseki et al., 2009). Taken together, this suggests both spatially and temporally differential theta-driven plasticity within the hippocampus.

Behavioral evidence in rats also supports a role of hippocampal theta activity during wake in memory encoding. Thus hippocampal theta power during encoding predicts the success of later recall (Berry and Thompson, 1978; Seager et al., 2002; Nokia et al., 2008), while disrupting theta activity pharmacologically—or through lesioning areas implicated in theta generation—significantly impairs learning (Winson, 1978; Givens and Olton, 1990). It appears not only the presence, but also the timing of learning with relation to theta is important in determining the success of encoding. Thus, the rate of learning in rabbits is fastest when hippocampal theta power is at its peak (Berry and Thompson, 1978). Also in rabbits, the rate of conditioning to a stimulus is increased in both delay (Seager et al., 2002) and trace conditioning (Griffin et al., 2004) when the stimulus is timed to appear during bouts/periods of the theta rhythm.

Human studies are comparatively scarce, given that hippocampal theta activity can only be recorded intracranially in epileptic patients. Whereas neocortical theta range (4–8 Hz) activity reliably predicts encoding, working memory and navigation (for a review, see Kahana et al., 2001), the link between hippocampal theta activity and memory appears to be more complex than that observed in rodents. Lega et al. (2012) were the first to systematically analyze hippocampal electroencephalography in humans during episodic memory encoding and retrieval. They reported peak activity around 3 and 8 Hz within the hippocampus. While the power of 3 Hz activity increased during successful encoding trials, 8 Hz activity displayed an inverse relationship. Furthermore, 3 Hz power was correlated with hippocampal gamma power. From this, the authors concluded that delta range 3 Hz activity within the hippocampus—similarly to the slower theta observed in humans during REM sleep—is the human analog to rodent hippocampal encoding-related theta activity. Both frequencies were synchronized between the hippocampus and the temporal cortex, suggesting hippocampal-cortical communication. Somewhat different results were reported by Rutishauser et al. (2010), who found that performance in a visual recognition task was predicted by spike coherence with ongoing hippocampal activity at an average of 5 Hz, though—similar to Lega et al. (2012)—this coherence peaked at 3 Hz.

Taken together, there is strong evidence supporting a role of theta activity during wake in the modulation of hippocampal plasticity which is clearly indicative of successful encoding/recall in behavior in both rodents and humans. The following section will highlight the similarities and differences between wake and REM sleep theta activity.

### Wake vs. REM Sleep Theta

Both wake and REM sleep theta share a similar frequency range and distribution throughout the brain in rodents and humans. Theta activity in both states is associated with the burst-like discharge of acetylcholine—which is strongly linked with plasticity—within the basal forebrain (Lee et al., 2005). There is however some evidence that wake and REM sleep theta differ in their generation and function. Firstly, a genetic mutation in mice was found to slow down hippocampal theta frequency (defined as 5–9 Hz) in REM sleep, but not in wake (Tafti et al., 2003). A further study found that coupling between theta and gamma activity within the parietal cortex of mice is greater in REM sleep compared to wake (Scheffzük et al., 2011). This particularly applies to the coupling between theta and fast gamma (120–160 Hz), which is ninefold stronger in REM vs. wake. Taken together, this suggests that—at least in rodents—wake and REM sleep theta differ in either their generation mechanism or regulation and may serve distinct, though possibly related functions.

### REM Sleep Theta and Gamma Coupling

Rapid-eye movement sleep theta activity appears to also modulate higher frequency activity in the brain. Thus, theta–gamma phase coupling during REM sleep within the hippocampal CA1 region in rats was found to be distinct for slow, mid-frequency and fast gamma (Belluscio et al., 2012). Gamma amplitude within these three bands was found to be modulated by theta phase. Phase-phase coupling was only detected between theta and slow and mid-frequency gamma, though not between theta and fast gamma. The authors interpreted this finding as suggesting an intricate multiple time-scale control of neuronal spikes during REM sleep, supporting information transfer and spike timing-dependent plasticity. Gamma oscillations nested within theta cycles have been proposed to allow the short term working memory of 7 ± 2 items in a list, as this number corresponds to the number of sub-cycles of gamma nested within one theta cycle (Lisman and Idiart, 1995). Therefore as different bands of gamma are coupled differentially to the phases of theta between wake and REM; REM sleep could effectively represent a reweighting of the items which are stored during wakefulness.

### Hippocampal Temporal Coding and Sequential Activity

A prominent feature of the hippocampus is the presence of neurons which fire in particular locations, with an ensemble forming a map of space when a rat navigates in the environment; these are termed place cells (O’Keefe and Dostrovsky, 1971). These cells have been found across several species including humans (Ekstrom et al., 2003). The spatial firing of these neurons is modulated by the environmental geometry (O’Keefe and Burgess, 1999), context (Anderson and Jeffery, 2003), and time/distance traveled (Pastalkova et al., 2008; MacDonald et al., 2011). This multi-faceted representation has led to the suggestion that they encode episodes structured in space or episodic memory on the single neuron level.

During wake, place cells are coordinated with the ongoing theta oscillation such that a place cell spikes at subsequently earlier phases of theta as the rat travels through the firing field of the cell (O’Keefe and Recce, 1993). Including and excluding spatially modulated cells, distinct classes of cells within the hippocampal formation (i.e., pyramidal, interneurons, and granule) fire on average at particular phases of theta (Skaggs and McNaughton, 1996). Skaggs and McNaughton (1996) noted that certain place cell firing within a theta cycle should reflect the order in which the cells’ place fields are arranged in space, thus giving rise the temporal encoding of traversed space over a theta cycle. Furthermore individual cells in the hippocampus fire at different phases of theta in novel vs. familiar environments in wake (Lever et al., 2010) and in REM sleep (Poe et al., 2000). Hasselmo (1999) proposed this phasic difference can act as a switch between encoding and retrieval of memories during wake. In REM sleep however, such a phase shift of cell firing could prioritize novel or emotionally salient memories while offloading the hippocampus of memories with less novelty or emotional salience. A possible indicator of such reorganization is provided in the work of Grosmark et al. (2012)—where a change in excitability is seen in the form of greater synchrony and decreased firing rate variability in the hippocampus following REM sleep with high theta power.

A specific phenomenon—initially termed replay—identified the re-activation of place cell sequences in a temporal order strikingly similar to that observed during previous experience (Dragoi and Buzsáki, 2006; Foster and Wilson, 2007; Gupta et al., 2012; O’Neill et al., 2010). This temporally coordinated firing of place cells takes place at a 20-fold faster rate than experienced during traversal of the environment. These events occur in both quiet wake (Foster and Wilson, 2006; Jackson et al., 2006; Diba and Buzsáki, 2007), and SWS—in both states these replay events are associated with the occurrence of hippocampal sharp-wave-ripples (SWRs; Wilson and McNaughton, 1994; Skaggs and McNaughton, 1996; Nadasdy et al., 1999; Lee and Wilson, 2002).

Temporally coordinated activity of place cells preceding experience in a novel unexplored environment (Dragoi and Tonegawa, 2011) has also been demonstrated, this phenomenon occurs before experience of the environment and therefore could underlie prospective planning by rehearsing and strengthening possible future trajectories.

Pertinent to our discussion, these sequences could also encode emotional salience, as reactivations leading to remembered goal (reward) locations appear to be preferentially activated during wake SWRs (Pfeiffer and Foster, 2013). Sequences associated with previously unexplored rewarded vs. unrewarded goal locations are also preferentially activated during prior SWS (Ólafsdóttir et al., 2015).

Critically, coordinated sequential activity of hippocampal place cells also occurs during REM sleep (Louie and Wilson, 2001) and thus in the presence of theta and absence of SWRs. Reactivations in REM sleep occur at the same timescale as wake—unlike the ∼20-fold increases seen in SWS. Interestingly, REM sleep appeared to only reactivate sequences previously activated during exploration of a familiar track, though not sequences associated with a novel environment. Although the sample sessions of REM sleep analyzed were few (15 REM episodes); this could be due to lower quality post-behavioral sleep following novelty. Similarly, recent evidence revealed coordinated sequential activity amongst head-direction cells (another spatial cell which fires when the animal’s head faces a particular allocentric direction) during REM sleep following wake experience also at a similar timescale to awake (Peyrache et al., 2015). These pieces of evidence clearly implicate the role of REM sleep in recapitulating wake experiences in terms of the sequential firing of individual neurons.

Though there are no human studies investigating sequential reactivation of place cells during REM sleep, the phenomenon of cued-memory reactivation during sleep (both during SWS and REM sleep) supports the idea that memories are reactivated during both sleep stages. Thus, sound cues associated with Morse code presented during REM sleep resulted in improved performance following sleep, though only if cueing occurred during phasic—not tonic (the distinction is elaborated further on)—REM sleep (Guerrien et al., 1989). In a separate study, participants were exposed to a loud ticking alarm clock while learning a set of complex rules (Smith and Weeden, 1990). Exposure to the same sound during following REM sleep led to a significant improvement in performance at a 1 week follow-up test compared to a non-cued group.

Sequential reactivations of cell assemblies representing experienced space are a clear candidate for stored memories. The fact that these occur during REM sleep – in coordination with ongoing theta activity – and that these reactivations are biased by exposure to memory cues during REM sleep – provides a strong case for REM sleep having a role in the processing of these memories.

### REM Sleep Theta and PGO Waves

Rapid-eye movement sleep theta activity appears to share a common generation mechanism with other prominent REM sleep features. Thus, the generation of REM sleep theta, REMs, REM sleep atonia and pontine-geniculo-occipital (PGO) waves all depend on the ventral part of the oral pontine reticular nucleus (vRPO; Reinoso-Suárez et al., 2001). PGO waves are large (250 mV) field potentials which propagate from the pontine tegmentum, to the lateral geniculate nuclei of the thalamus and the occipital cortex (Nelson et al., 1983; Callaway et al., 1987). PGO waves during REM sleep in rodents have been repeatedly linked with emotional memory consolidation. While suppressing PGO wave generation in rats impaired avoidance memory retention across sleep (Mavanji et al., 2004), artificially enhancing PGO waves through injecting carbachol prevented deficits in avoidance memory across a period of REMSD (Datta et al., 2004). It appears the quality of PGO wave activity is directly related to memory processes, thus a number of studies have reported an increase in PGO wave density following fear memory training in rats which predicted overnight strengthening of the memory (Datta, 2000; Datta and Saha, 2005; Ulloor and Datta, 2005; Datta et al., 2008). Additionally, the success of fear extinction was recently shown to be predicted by PGO wave quality during REM sleep (Datta and O’Malley, 2013). Furthermore, post-training PGO wave density was associated with increased activity of brain-derived neurotrophic factors and plasticity-related immediate early genes in the dorsal hippocampus (Ulloor and Datta, 2005; Datta et al., 2008). Selective elimination of PGO wave generating cells prevented these increases, while enhancing PGO waves through cholinergic activation of these cells augmented the increases. Thus, it has been proposed that PGO waves enhance synaptic plasticity in areas they pass through (Datta et al., 2011), which includes the hippocampus and amygdala (Datta et al., 1998).

Besides having an overlapping generation mechanism, PGO waves and REMs tend to be phase-locked to theta waves (Karashima et al., 2001, 2004, 2007). It appears both are driven by theta oscillations. Thus, eliciting theta activity in REM sleep through electrical stimulation applied to the medial septum in rats, entrains PGO waves and REMs to theta (Reinoso-Suárez et al., 2001). Conversely, when PGO waves are elicited through audio stimulation, theta phase is not reset, though PGO waves eventually become phase locked with theta again (Karashima et al., 2002). In a further study, Karashima et al. (2004) reported a positive correlation between both peak frequency and amplitude of hippocampal theta oscillations with PGO wave and REM density in rats. The same group also found that in both cats and rats, theta frequency accelerates several 100 ms prior to the negative peak of PGO waves (Karashima et al., 2005). When PGO waves are inhibited by lesions to the subcoeruleus region, where PGO waves are generated, synchronization between regional theta waves is disrupted. Critically, PGO wave density also reflects theta synchronization between the hippocampus and amygdala during REM sleep in rats (Karashima et al., 2010).

Though readily detectible in rats and cats, scalp EEG does not suffice in revealing PGO activity in humans. However, based on fMRI evidence, the pontine tegmentum, thalamus, primary visual cortex, putamen, and limbic areas activate in synchrony with the occurrence of REMs, which strongly suggests a similar activity in humans (Wehrle et al., 2007; Miyauchi et al., 2009). Due to the common generation mechanism of REMs and PGO waves, REM density is a commonly used measure for REM sleep intensity in humans. REM density has been observed to increase following stressful periods of learning—based on University students during an exam preparation (Smith and Lapp, 1991). This could reflect the enhanced processing of emotional memories (i.e., exam material paired with the fear of failing an exam) during REM sleep.

Taken together, it appears that in rodents, theta activity during REM sleep—and specifically the theta synchronization between the amygdala and hippocampus—drive large scale synchronized activation in the form of PGO waves, which in turn enhance synaptic plasticity within the hippocampus and amygdala. This, in combination with the evidence linking PGO wave density with emotional memory processing, strongly supports a specific role of REM sleep theta activity in the selective processing of emotional memories. It is possible that a similar relationship between theta coherence within the limbic system and PGO waves exists in humans, however evidence for this is lacking.

### The Role of REM Sleep Theta in Emotional Memory

In addition to evidence linking the occurrence of REM sleep with emotional memory consolidation, there is some evidence directly linking REM sleep theta with emotional memory processing. Following training in an avoidance task, compared to a preceding control night, rats displayed an increased amount of REM sleep periods 17–20 h following training, as well as increased theta power during these REM sleep periods (Fogel et al., 2009). Interestingly, sleep spindle activity during SWS was also enhanced, supporting the notion that the two sleep stages serve complimentary roles in fear memory processing. In a study by Hegde et al. (2011) REM sleep duration in rats was also increased during the recovery period following several days of chronic stress immobilization. The authors also found a decrease in theta coordination between the hippocampus and lateral amygdala which was negatively correlated with REM sleep duration.

A more compelling link between REM sleep theta and fear memory consolidation was shown in a study on rats by Popa et al. (2010), which showed that theta coherence between the hippocampus, medial prefrontal cortex and amygdala predicted bidirectional changes in fear memory across sleep. These same brain areas exhibit synchronized theta activity when a rat is presented with a conditioned stimulus in wake following Pavlovian fear learning (Seidenbecher et al., 2003). This is observed for long term conditioning (24 h following fear conditioning), however not in the short term (within 2 h; Narayanan et al., 2007). This supports the notion that coherent theta activity within these areas during REM sleep is somehow involved in the long term consolidation of fear memories.

Due to the lack of an adequate non-emotional control task in rodent studies, we cannot exclude the possibility that non-emotional memory consolidation is equally dependent on REM sleep theta activity. Human studies allow a clear separation of emotional and non-emotional memories—though human intracranial studies investigating the role of hippocampal theta activity during REM sleep on emotional memory processing are limited. However, given the coherence of theta activity between the hippocampus, amygdala and neocortex in rats both during wake (Siapas et al., 2005; Lesting et al., 2011, 2013) and REM sleep (Popa et al., 2010) it is possible that a similar coherence exists in humans. Theta activity recorded from the scalp during REM sleep using EEG may therefore be indicative of hippocampal theta activity. One such EEG based study in humans found that right prefrontal REM sleep theta (4–7 Hz) power during an afternoon nap predicted superior post-nap recognition of emotionally negative vs. neutral images encoded before the nap (Nishida et al., 2009). In a wake control group, recognition of both image types did not exceed pre-nap baseline levels. In a separate study, Prehn-Kristensen et al. (2013) compared sleep-dependent emotional memory consolidation between adults, healthy children and children with ADHD. They discovered a stronger emotional benefit in healthy children compared to both other groups. Similarly to the previous study, frontal theta activity positively correlated with emotional memory performance in both adults and healthy children, however in children with ADHD, frontal theta activity negatively predicted emotional memory performance. Based on these two studies, it appears cortical theta activity during REM sleep represents some form of emotional memory processing in humans. However, intracortical evidence is needed to clarify the role of the hippocampus in this process.

### REM Sleep Theta During Tonic vs. Phasic REM Sleep

Rapid-eye movement sleep itself is not a homogenous state. It is often described as either phasic or tonic—with tonic REM sleep taking up approximately 95% of REM sleep in rats (Montgomery et al., 2008). During phasic REM sleep, REMs, PGO waves and muscle twitches occur in bursts and are accompanied by an increase in vegetative activation as well as an increase in both frequency and amplitude of hippocampal theta activity (Brankačk et al., 2012). Tonic REM sleep in contrast is characterized by more evenly distributed PGO waves.

The existence of phasic REM sleep in humans is assumed based on a similar occurrence of phasic bursts of REMs accompanied by muscle twitches and cardiorespiratory irregularities (Carskadon and Dement, 2005). Interestingly, unlike in rodents in which hippocampal theta activity occurs throughout REM sleep (Brankačk et al., 2012), Cantero et al. (2003) described phasic bursts of REM sleep theta activity in humans—approximately 1 s in duration—and a lack of theta activity during tonic REM sleep. A previous study had failed to detect significant REM sleep theta activity during human REM sleep within the human hippocampus (Halgren et al., 1978; Bódizs et al., 2001), while a further study reported only a rare occurrence of theta in cortices surrounding the hippocampus (Uchida et al., 2001, 2003). Discrepancies between these studies were ascribed to differences in recording methods, specific brain areas recorded from, and the pathology of the respective subjects (e.g., epilepsy; Bódizs et al., 2005; Tamura et al., 2013).

In rats, theta and gamma synchrony within the hippocampus are increased during phasic vs. tonic REM sleep (Montgomery et al., 2008). During tonic REM sleep, theta coherence is increased within the dentate gyrus (DG) and between the DG and CA3. Montgomery et al. (2008) suggest that the increase in DG synchrony accompanied by CA1/CA3 coherence reduction in the gamma range during tonic REM could mediate pattern separation for subsequent retrieval, which is thought to be a key function of the DG during wakefulness (Leutgeb et al., 2007; McHugh et al., 2007; Bakker et al., 2008). The heightened acetylcholine levels during REM sleep would facilitate this pattern separation by reducing interference of excitatory feedback within the hippocampus (Hasselmo, 1999), thus facilitating selective encoding.

In contrast, phasic REM sees an increase in theta and gamma coherence in DG together with an increase in gamma coherence in CA3/CA1, thus facilitating greater information exchange between the hippocampus and neocortex during REM sleep (Datta et al., 2004; Karashima et al., 2005; Montgomery et al., 2008), in which activity could be replayed to the cortex (Louie and Wilson, 2001). A separate study found greater theta synchrony within the prefrontal cortex of rats during phasic vs. tonic REM and an increased theta/gamma synchrony within the prefrontal cortex during tonic vs. phasic REM sleep (Brankačk et al., 2012). Though the significance of these findings in linking the two REM sleep states with memory is yet unclear, they support the notion that they serve separate functions.

## Conclusion

Based on the evidence reviewed above, it appears that theta activity during REM sleep drives the emotionally modulated processing of novel memory traces within the hippocampus, possibly in preparation for consolidation during NREM sleep. This is supported by the observation that theta activity—in particular theta coherence between the hippocampus and amygdala—drives high-amplitude PGO waves, the density of which predicts the expression of plasticity-related genes within the hippocampus and amygdala. As amygdala-hippocampal coherence during wake predicts future recall of emotional events, it is possible that this coherence in REM sleep reflects the selective processing of emotional memory traces.

The timing of spike activity within the hippocampus with respect to theta phase is vital in determining whether long term potentiation (LTP) or depression (LTD) occurs. While hippocampal activity associated with a novel experience occurs at a similar phase during REM sleep compared to wake—thus promoting LTP—activity associated with a familiar experience occurs at a phase promoting LTD. In addition, evidence suggests theta activity during REM sleep increases unit synchrony and decreases firing rate variability. Taken together, this implies REM sleep in processes of separation and offloading of traces previously consolidated during NREM sleep, thus freeing capacity to encode and process novel memories.

In addition to emotionally modulated memory processing within the hippocampus, theta activity during REM sleep could also mediate the integration of novel memory traces within the neocortex. Acetylcholine—which reduces hippocampal interference in the neocortex—is raised during REM sleep and directly correlated with theta activity within the hippocampus. In addition to the rise in acetylcholine, noradrenaline levels are extremely low during REM sleep. As noradrenaline suppresses recurrent activation within the neocortex, this drop in noradrenaline during REM sleep could allow encoding-like processes of a more elaborate nature than wake, i.e., allowing recombination and integration of novel memory traces into pre-existing memory networks. This neocortical integration may give rise to the vivid dreaming experienced during REM sleep.

Those who refute a role of REM sleep in memory (Vertes and Eastman, 2000; Siegel, 2001) refer to the observation that many antidepressants markedly reduce REM sleep in humans without causing significant deficits in learning or memory consolidation (Vertes and Eastman, 2000). Instead, Vertes and Eastman (2000) suggest the primary function of REM sleep is to provide a period of wake-like endogenous stimulation, allowing the brain to maintain necessary levels of activity throughout sleep. By doing so, REM sleep supposedly promotes the recovery from SWS and thus prepares the brain for wakefulness. However, if this was the case, one would expect the amount of REM sleep in each sleep cycle to correlate with the respective amount of SWS. Instead, REM sleep displays an inverse relationship with SWS in humans (and remaining constant irrespective of SWS in rodents) across sleep. This suggests that the function of REM sleep may not be merely a reversal of processes in SWS, but potentially an extension of SWS-dependent processes—such as the integration of novel neocortical representation within existing memory networks.

This emotionally modulated processing of memories within the hippocampus may prioritize the consolidation of emotional memories during NREM sleep. The following integration of these novel memories could allow emotional experiences to guide future behavior in similar situations. This is backed by behavioral evidence, which associates REM sleep with the selective strengthening of emotional memories and the modulation of emotional responses. The REM sleep dependent bidirectional changes in arousal reported in the literature could also be explained by integratory processes during REM sleep: if an emotional response associated with a given experience is of no direct use to the memory itself, this may be lost in this process. In contrast, an emotional response which is integral to the memory (e.g., avoidance of a deadly animal), the emotional response may be strengthened along with the memory. Thus the main function of REM sleep may be the emotionally modulated optimization of behavior.

This proposed function of REM sleep would explain why the suppression of REM sleep does not cause noticeable cognitive deficits in patients using REM-suppressing antidepressants. If not faced with emotionally trying experiences or significant changes to the environment—which would require rapid adaptation—the patients can rely on “outdated” behavioral responses to similar situations. Therefore, we hypothesize that REM sleep suppression during early childhood development would result in more striking behavioral deficits. In addition we hypothesize that parametrically varying emotional weight in the form of rewards/punishments in behavioral tasks should change the degree of theta synchronization between limbic structures, distribution of theta-phase firing of individual cells and also manifest itself as a differential number of sequence reactivations during REM sleep. Despite behavioral evidence predominantly pointing toward a role of REM sleep in predominantly emotional memory, it remains unclear whether non-emotional memories are processed independently of REM sleep—it is possible that they are also processed during REM sleep though with a lower priority. Further, it would be interesting to consider how the previously alluded to evidence linking REM sleep and procedural memory ties in with the proposed role of REM in emotional memory processing.

### Conflict of Interest Statement

The authors declare that the research was conducted in the absence of any commercial or financial relationships that could be construed as a potential conflict of interest.
